# Progress and Challenges in the Improvement of Ornamental Plants by Genome Editing

**DOI:** 10.3390/plants9060687

**Published:** 2020-05-28

**Authors:** Chang Ho Ahn, Mummadireddy Ramya, Hye Ryun An, Pil Man Park, Yae-Jin Kim, Su Young Lee, Seonghoe Jang

**Affiliations:** 1 Floriculture Research Division, National Institute of Horticultural and Herbal Science, Rural Development Administration (RDA), Wanju-gun, Jellabuk-do 55365, Korea; dosanahn@gmail.com (C.H.A.); ramya87.4u@gmail.com (M.R.); hryun@korea.kr (H.R.A.); pmpark@korea.kr (P.M.P.); yj0503@korea.kr (Y.-J.K.); 2 World Vegetable Center Korea Office (WKO), Wanju-gun, Jellabuk-do 55365, Korea

**Keywords:** CRISPR, genome editing, ornamental plant, plant transformation, precision editing

## Abstract

Biotechnological approaches have been used to modify the floral color, size, and fragrance of ornamental plants, as well as to increase disease resistance and vase life. Together with the advancement of whole genome sequencing technologies, new plant breeding techniques have rapidly emerged in recent years. Compared to the early versions of gene editing tools, such as meganucleases (MNs), zinc fingers (ZFNs), and transcription activator-like effector nucleases (TALENs), clustered regularly interspaced short palindromic repeat (CRISPR) is capable of altering a genome more efficiently and with higher accuracy. Most recently, new CRISPR systems, including base editors and prime editors, confer reduced off-target activity with improved DNA specificity and an expanded targeting scope. However, there are still controversial issues worldwide for the recognition of genome-edited plants, including whether genome-edited plants are genetically modified organisms and require a safety evaluation process. In the current review, we briefly summarize the current progress in gene editing systems and also introduce successful/representative cases of the CRISPR system application for the improvement of ornamental plants with desirable traits. Furthermore, potential challenges and future prospects in the use of genome-editing tools for ornamental plants are also discussed.

## 1. Introduction of Genome-Editing Technologies

Floricultural crops are an economically fundamental part of horticulture production systems. In particular, the major types of cut flowers, such as carnations, chrysanthemums, roses, and tulips, play a crucial role in the horticultural industry because of their aesthetic importance. For a long time, ornamental plants possessing no nutritive value have been cultivated entirely for the ornamental value of their flowers. Consumers demand novel cultivars with new morphologies, colors, and fragrances, and are eager for new types of ornamental plants; therefore, the lifespan of ornamental plant cultivars in commercial floriculture is much shorter than that of other crops [1]. Till now, to produce new cultivars of ornamental plants, various breeding strategies have been used. Among them, conventional breeding techniques, such as hybridization and mutation breeding technologies, have long been employed, and these techniques have been widely used to produce various colors and shapes, as well as improve plant architecture and disease resistance. However, these conventional methods have several limitations and drawbacks for ornamental plants because many of them are highly heterozygous, which causes a complex inheritance of genetic factors as well as polyploidy [2,3]. Thus, alternative approaches are required for further improvement of ornamental plant production.

In the last few decades, genome sequencing technology has been vastly and rapidly improved. In particular, after the advent of next-generation sequencing technology in 2005, genomic sequence information on major ornamental plant species, including *Chrysanthemum nankingense* [4], *Dendrobium catenatum* [5], *Dendrobium officinale* [6], *Dianthus caryophyllus* [7], *Helianthus annuus* [8], *Hibiscus syriacus* [9], *Ipomoea nil* [10], *Petunia hybrida* [11], *Phalaenopsis* [12,13], *Primula veris* [14], *Rosa chinensis* [15,16], *Rosa multiflora* [17], and *Rosa roxburghii* [18], has been reported in just a few years. Given the increasing number of ornamental plant genomes that have been sequenced, the information will be of great help in the breeding of ornamental plants, as well as for basic research. Genetic transformation is a powerful tool and could be useful in producing an “additive” one-point improvement compared to mutation breeding, which produces a “subtractive” one-point improvement [19]. Since the first report of genetic transformation in ornamental plants in 1987 [20], genetic transformation has been recognized as an important technique for generating new cultivars. Despite at least 50 ornamental plants being transformed [21], very few genetically modified (GM) ornamental plants have been approved and commercialized, and only in a few countries. For instance, both a violet carnation, “Moondust™”, and a blue rose, “Applause™”, which are GM to accumulate delphinidin-based anthocyanins, were generated and commercialized by the Australian biotechnology company Florigene and the Japanese company SUNTORY, respectively, in the global flower market. Even though GM ornamental plants are likely to be more acceptable to consumers than GM food crops [22], GM plants developed using recombinant DNA technology are strictly regulated in many countries based on biosafety or risk assessment issues [23].

In parallel to the classic GM techniques (e.g., gene overexpression and RNA silencing), zinc-finger nucleases (ZFNs), the first generation of genome-editing tools, initially emerged in 1996 and have been applied to a wide variety of organisms. However, the application of ZFNs requires not only the proper design and assembly of new ZFNs, but also the validation of their activity through a time-consuming screening process [24]. Transcription activator-like effector nucleases (TALENs) have been reported to be an alternative to ZFNs for genome editing. Compared to ZFNs, especially, TALENs turned out to be much easier to design [25]. Currently, new genome-editing technologies, such as the clustered regularly interspaced short palindromic repeats–CRISPR-associated 9 (CRISPR-Cas9) and CRISPR from *Prevotella* and *Francisella* 1 (CRISPR-Cpf1), have been developed for the genetic modification of organisms, including ornamental plant species. Unlike ZFNs and TALENs, the CRISPR system is an RNA-directed DNA endonuclease adapted from the bacterial immune system. CRISPR provides several advantages in terms of simple design and cost over ZFNs and TALENs [26,27]. In this review, we summarize the engineered nucleases that have been used for genome editing and also consider their current applications in ornamental plants. Finally, we discuss the challenges of genome editing for the production of ornamental plants with desirable/better traits.

## 2. Genome-Editing Technologies

### 2.1. Zinc-Finger Nucleases (ZFNs) and Transcription Activator-Like Effector Nucleases (TALENs)

ZFNs are the first generation of engineered endonucleases and artificial fusion proteins that connect a zinc finger DNA binding domain to a non-specific DNA cleavage domain of the FokI restriction endonuclease. Unlike in other restriction enzymes, in FokI, the type II restriction endonuclease consists of an N-terminal DNA-binding domain and the non-specific DNA cleavage domain at the C-terminal only functions as a dimer [28]. Each zinc-finger recognizes a 3-bp DNA sequence. ZFNs are designed as two ZFN monomers bound to an 18–24-base pair sequence with a 5–7-nucleotide spacer [29]. Since the first report published in 1996, many studies of ZFNs have been successfully applied to gene modification in plants, such as *Arabidopsis*, tobacco, and maize [30]. Over the last two decades, various methods have been developed to improve the applicability, efficiency, and precision of ZFNs. However, there are still concerns about interference from neighboring finger domains and the limited number of recognition sites [31], although target gene modification via ZFNs has been reported in various plants, including ornamental plants (*Petunia hybrida*) [28].

Similar to ZFNs, TALENs are also composed of a DNA-binding domain and a FokI nuclease domain. The applications of TALENs are based on the recognition of the functional principles of the transcription activator-like (TAL)-type III effectors that are derived from the plant pathogenic bacteria *Xanthomonas* [30]. After being transferred into host cells, TAL effectors enter into the nucleus and bind to target gene promoters within 60-bp of start codons and activate transcription. The DNA-binding domain of the TAL effector is prefabricated to recognize DNA sequences, similar to zinc-finger proteins; however, unlike the ZFNs system, the TAL effector domain consists of 33–35 amino acid residues and recognizes one base pair per unit. The binding specificity of the TAL effectors is determined by two hypervariable amino acids, known as repeat variable di-residues (RVDs), located at the 12th and 13th position in each repeat [32]. DNA binding preference by each repeat is determined by only two RVDs. In other words, the four most common RVDs—HD, NG, NI, and NN—preferentially recognize C, T, A, and G/A bases, respectively. Therefore, it is possible to manufacture TAL effectors that can bind to any DNA sequences by basically only four TAL effector modules. Compared to ZFNs, TALENs have high success rates based on the ease of design, better binding affinity to their target sites through RVDs, and a higher level of cleavage activity. However, working with ZFNs or TALENs is expensive and laborious.

### 2.2. CRISPR-Associated Systems

The CRISPR genome-editing system was developed about two years after the discovery of TALEN proteins [33]. The CRISPR-Cas system was originally derived from the immune systems of prokaryotes, such as bacteria and archaea [34]. The genomic CRISPR locus contains the cas operon, the CRISPR repeat-spacer array, and a sequence located upstream of the cas operon coding for a non-protein coding trans-activating RNA [35]. The CRISPR system differs from ZFNs and TALENs in that the Cas protein, which has nuclease activity, cuts DNA instead of the restriction enzyme FokI. In other words, the CRISPR system depends on RNA–DNA binding to determine specificity, while the ZFN and TALEN systems rely on protein–DNA binding for target sequence recognition. CRISPR-Cas systems can be functionally divided into two classes. The Class 1 systems are characterized by multi-subunits of effector nuclease complexes while the Class 2 systems consist of a single protein multi-domain effector, such as Cas9. The two classes are subdivided into six types: type I, III, and IV for Class 1; and type II, V, and VI for Class 2 [36]. Of the three types, type II CRISPR-Cas systems have been identified for use in genome editing. Type I and type III are composed of several Cas proteins, whereas the Cas9 protein is the only nuclease protein constituent of type II CRISPR-Cas systems [37].

**Figure 1:**
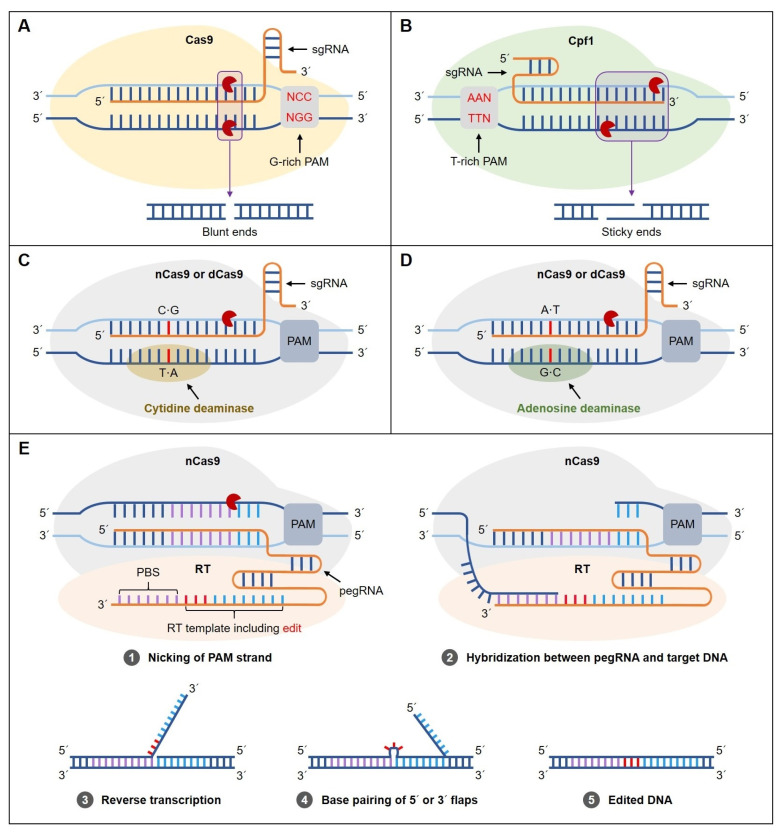
Clustered regularly interspaced short palindromic repeat (CRISPR) systems. (**A**) In the CRISPR–CRISPR-associated 9 (CRISPR-Cas9) system, a G-rich protospacer adjacent motif (PAM) creates double-stranded breaks (DSBs) toward the proximal end of the recognition site resulting in blunt ends. (**B**) In the CRISPR-Cpf1 system, a T-rich PAM creates DSBs at the distal region of the recognition site, producing sticky ends. (**C**) A CRISPR-Cas9-mediated cytosine base editor (CBE) system. The Cas9 nickase (nCas9) or catalytically dead Cas9 endonuclease (dCas9) fused to the Cas9–cytidine deaminase domain is guided by a single-guide RNA (sgRNA) to make single-base changes. The Cas9–cytidine deaminase makes the base change (C·G-to-T·A conversion). (**D**) A CRISPR-Cas9-mediated adenine base editor (ABE) system. The nCas9 or dCas9 fused to the Cas9–adenosine deaminase domain is guided by a sgRNA to make single-base changes. As with the CBE system, A·T-to-G·C conversion is achieved at a target site. (**E**) Prime-editing system. Gray circles with white numbers indicate the order of the editing process. (1) The prime editor consists of nCas9 fused to a reverse transcriptase (RT) and prime-editing guide RNA (pegRNA) bind to target DNA; (2) the nicked strand hybridizes the prime binding site (PBS) on the extended 3′ end of the pegRNA; (3) the RT extends the nicked DNA strand; (4) the extended strand competes for binding to the target DNA; and (5) DNA repair results in edited DNA.

Cas9, the effector protein of type II CRISPR-Cas systems, can be targeted to a specific genomic sequence by approximately 20 nucleotides of the single-guide RNA (sgRNA). DNA cleavage only occurs when a G-rich (5′-NGG-3′, where N = A, T, C, or G) protospacer adjacent motif (PAM) is identified downstream of the DNA target site (Figure 1A). Recently, a new Cas protein named Cpf1, also known as CRISPR-Cas12a, has been found to be efficient in plant genome editing. In contrast to Cas9 proteins recognizing G-rich PAM sequences, Cpf1 recognizes T-rich (5′-TTTV-3′, where V = A, G, or C) PAM sequences (Figure 1B). Moreover, Cpf1 does not require an additional transactivating CRISPR RNA (tracrRNA) to form a mature crRNA. Non-homologous end joining (NHEJ) is the most common mechanism in plant cells employed to repair double-stranded breaks (DSBs) [26]. Thus, homologous recombination (HR) repair via exogenously supplied donor DNA is not so efficient, limiting its application in plants. Consequently, base editing (as well as prime editing) represents an alternative tool to homology-directed repair (HDR)-mediated replacement, facilitating precise editing without a DSB and a donor repair template. Although CRISPR-Cas9 systems are suitable for gene knock-out or knock-in, they cannot convert one base into another [38]. Single-base changes can contribute to promoting plant improvement. Recently, single-base-editing tools, such as cytosine base editors (CBEs; targeted C·G to T·A) and adenosine base editors (ABEs; targeted A·T to G·C), have been developed and exploited to induce targeted base conversion in model plants, such as rice and *Arabidopsis*. These tools induce base changes without double-strand breaks through a fusion of adenine/cytidine deaminase to either a Cas9 nickase (nCas9) or catalytically inactive Cas9 (dead Cas9/dCas9) [39,40]. Without harboring selection markers, herbicide-resistant tomatoes containing multiple point mutations were generated using CBEs by editing the *acetolactate synthase* (*ALS*) gene [41], and the functional activity of ABEs enabling A·T-to-G·C conversion was also verified in rice and wheat plants. Especially, modified sequences of the *Acetyl-coenzyme A carboxylase* (*ACC*) gene in mutant rice plants were confirmed together with their herbicide-tolerant phenotype [42]. Consequently, fusions, such as the Cas9–cytidine deaminase and Cas9–adenosine deaminase, can convert one base to another (four transition mutations: C→T, G→A, A→G, and T→C) (Figure 1C,D). However, base-editing systems also have limitations, such as transversions, insertions, or deletions. Prime editing, a new method to overcome such problems, has been recently reported by Anzalone et al. [43]. This system offers greater editing precision without any double-strand breaks or donor DNA templates compared to other CRISPR alternatives. The first part of the prime-editing guide RNA (pegRNA) provides a sequence to detect a specific target site and Cas9 finds it and cuts a single-strand DNA. Then, a reverse transcriptase (RT) synthesizes DNA from the second part of the pegRNA, including corrections, insertions, or deletions. Prime editors, which are fusions of the Cas9 nickase (nCas9) and RT, enable us to induce the four transition and all eight transversion mutations (C→A, C→G, G→C, G→T, A→C, A→T, T→A, and T→G; Figure 1E). A comparison of the CRISPR genome-editing features between the base editor and prime editor is presented in Table 1. To date, the base-editing system has been reported in crops such as rice, wheat, maize, and tomato [42,44,45,46], and the prime-editing system has been successful in rice and wheat [47]. However, neither the base-editing nor prime-editing approach has yet been reported for inducing mutations in ornamental plants.

## 3. Current Status of Genome Editing in Ornamental Plants

In recent years, the manipulation of precise genome editing with efficient targeting has become possible in plants. Genomic sequence information analysis technology is rapidly being developed with higher accuracy and, subsequently, a large amount of bioinformatic data has been released for major crop plants. As described above, various genome-editing systems have been reported, and the development of the CRISPR-Cas system is anticipated to create new possibilities for the breeding process of ornamental plants. In ornamental plants, many studies have attempted to improve floricultural traits, including floral color, size, shape, fragrance, disease resistance, vase life, and so on. However, since it is not easy to apply genome-editing systems to ornamental plants whose genome sequence information remains unknown, only a few studies using CRISPR-Cas genome-editing systems have been reported in ornamental plants. Moreover, higher polyploidy ornamental plant species, such as roses (tetraploid) and chrysanthemums (hexaploid), require a highly efficient editing platform for the future improvement of these plants. Therefore, more accurate information on whole-genome sequencing should be improved. From previously published research articles, 11 scientific publications related to the genome editing by CRISPR-Cas systems in ornamental plants have been collected (Table 2). Petunias are economically important and have gradually become increasingly popular worldwide. The first ornamental plant genome to be edited by the CRISPR-Cas9 system was that of *Petunia hybrida*. Subburaj et al. [48] demonstrated an efficient targeted mutagenesis in the *NITRATE REDUCTASE* (*PhNR*) gene by the direct delivery of engineered RNA-guided endonuclease (RGEN) ribonucleoproteins (RNPs) into the protoplast cells of *Petunia*, and Zhang et al. [49] also reported the successful generation of mutations at desired positions on the *Petunia phytoene desaturase* (*PhPDS*) gene, resulting in an albino phenotype as expected. Sun and Kao [50] utilized the polycistronic tRNA–gRNA (PTG)-based CRISPR-Cas9 genome-editing system [51] with a view to generate frame-shift indel alleles of *PiSSK1* (encoding the Skp1 subunit of SCF E3 ubiquitin ligase complex) in *Petunia inflate*. The PTG-based CRISPR-Cas system was developed for generating multiple gRNAs from a single polycitronic gene (for multiplex editing) via the endogenous tRNA-processing machinery that precisely cleaves both ends of the tRNA precursor [51]. The authors showed that a complete absence of *PiSSK1* in transgenic pollen grains inhibited pollen tube growth; this inhibition was a typical self-incompatibility response. Most recently, a key gene encoding the ethylene biosynthesis enzyme *1-aminocyclopropane-1-carboxylate oxidase1* (*PhACO1*) was edited in the *Petunia* cultivar “Mirage Rose” using CRISPR-Cas9 [52]. Transgenic *Petunia* plants exhibited reduced ethylene production with delayed petal senescence. Delayed petal senescence was also observed in mutant Japanese morning glory (*Ipomoea nil* “Violet”) plants generated by the CRISPR-Cas9 system that targeted the *EPHEMERAL1* (*EPH1*) gene, encoding the master regulator of petal senescence as an NAC transcription [53]. Flower color is one of the most important commercial traits in ornamental plants [21], and it is derived mainly from flavonoids, carotenoids, and betalains [54]. Currently, flower color modification has been widely applied using genome-editing tools in many plant species. The first floral color modification in higher plants by CRISPR-Cas9-mediated mutagenesis was achieved by the manipulation of the *dihydroflavonol-4-reductase* (*DFR*) gene in *Ipomoea nil* [55]. In 2018, Watanabe et al. [56] produced mutant *Ipomoea nil* plants with pale-yellow petals through CRISPR-Cas9-mediated mutagenesis on the *carotenoid cleavage dioxygenase 4* (*CCD4*) gene. In addition, Nishihara et al. [57] observed a color change from pale blue to white in *Torenia fournieri* through CRISPR-Cas9-mediated mutation on the *flavone 3-hydrolase* (*F3H*) gene, which encodes a key enzyme in the flavonoid biosynthetic pathway. Two genetic transformation systems, such as somatic embryogenesis and adventitious bud regeneration, have been established for the application of CRISPR-Cas9 technology in two *Lilium* species, *L. longiflorum* and *L. pumilum*. By targeting the *phytoene desaturase* (*PDS*) gene encoding a key enzyme of carotenoid synthesis for induced mutagenesis, mutant *Lilium* species of the albino, pale-yellow, and albino-green chimeric types were produced with various patterns of mutation, including base insertion, deletion, and substitution [58]. In addition, some results from orchid studies using CRISPR-Cas systems for genome editing have been recently released. Although plants belonging to the Orchidaceae, the second largest family of flowering plants, currently occupy an important position in global commercial floriculture crop production based on economic value [59], limited genomic sequence information on orchid species is available: genome information is available for *Dendrobium catenatum* [5], *Dendrobium officinale* [6], *Gastrodia elata* [60], *Phalaenopsis aphrodite* [61], and *Phalaenopsis equestris* [12]. Moreover, only a couple of studies involving genome editing by CRISPR-Cas systems have been reported in orchids. Kui et al. [62] applied the CRISPR-Cas9-mediated genome-editing system in *Dendrobium officinale* and demonstrated that this system could generate edits at a rate of 10% to 100%. Roles of MADS-box genes in flower development have been intensively studied in a wide variety of plant species [63]. Recent studies have displayed that the CRISPR-Cas9 system has been successfully employed to create multiple mutants of MADS-box genes in the orchid *Phalaenopsis equestris* [64]. *Chrysanthemum*, a widely grown ornamental plant, is one of the most economically important and favored floricultural crops [65]. Even though its whole-genome sequence information has been released, highly specific genome editing by CRISPR-Cas systems in *Chrysanthemum* is likely to be difficult owing to its large genome size and higher genomic ploidy levels with a high content of repeated sequences. Currently, only a single report demonstrating that the CRISPR-Cas9 system is working in *Chrysanthemum* is available [66].

## 4. Challenges and Future Prospects of Genome Editing in Ornamental Plants

Genome-editing technology can be regarded as one of the methods for mutational breeding. TALENs, ZFNs, and mega nucleases (MNs) are early versions of gene-editing tools for genome manipulation. Recently, new CRISPR systems, including base editors and prime editors, have conferred reduced off-target activity with improved DNA specificity, expanding the targeting scope and thus resulting in being more robust and cost effective [67,68,69]. To date, several reports have been positively engaged in directed gene editing in models and major crop plants through the CRISPR-Cas9 system. While genome editing has numerous benefits compared to conventional crop breeding, some challenges still remain in floricultural crops.

In floricultural crops, molecular and genetic studies are challenging, which hinders the identification of responsible genes for key traits. Genome sequencing of floricultural crops of interest will be of great help in identifying the genes associated with desirable traits. Because of the lack of a reference genome, the aimed sequence could be cloned by reproducible designing primers for conserved protein motifs with putative functions related to appropriate traits. The easy approach to gene/genome editing signifies a vigorous tool not only for functional analysis of gene(s), but also for breeders in the integration of genetic factors into the genomes of economically important crops. The site-directed mutagenesis of various genes can provide key information on their functions. The simultaneous targeting of multiple genes/loci through multiplex strategies can promote studies to identify the roles of individual genes in the intracellular signaling pathways. The preferred CRISPR-Cas9 method can be exemplified in completely knocked-out gene function [70,71], microRNA knock-down screening [70], and the programmed editing of certain loci, providing the functional analysis of cis- and trans-regulatory elements/factors with high accuracy [70]. In addition, the CRISPR-Cas9 system can be applied in the formation of conditional alleles, providing spatial and/or temporal control of gene expression to study the function of lethal genes.

Additionally, the CRISPR-Cas system opens up wide opportunities for visualizing the endogenous gene expressions in vivo through applying fluorescence. Moreover, it can also be applied to the research on histone modification and DNA methylation. For example, utilizing inactive dCas9 as a DNA-binding domain fused enzymes such as DNA methylases, histone acetyltransferases, and deacetylases, and can be targeted to alter the epigenetic state at precise locations within the genome [72]. Phenotypic alterations with desirable traits caused by the modification of gene expression—knock-down through antisense or double-stranded RNA interference (dsRNAi) approaches and “gain-of-function” approaches for target genes—can also be valuable information for the application of gene editing technologies with versatility in further breeding. Furthermore, overexpression of transcription factors fused to chimeric repressors demonstrated loss-of-function phenotypes; i.e., multi-petal phenotypes were observed from the transgenic *Cyclamen persicum* plants harboring a chimeric repressor construct, *CpAG1-SRDX* or *CpAG2-SRDX*, for *AGAMOUS* (*AG*) orthologs with strong repression domains (SRDX) [73]. The choice of spatio-temporal or tissue-specific promoters (e.g., ovule specific) for the Cas9 expression could help to limit the effects of the mutation to specific organs and/or tissues improving the trait of interest, without generating pleiotropic effects. Recently, a miniature chrysanthemum with delayed flowering was generated through the dsRNAi approach targeting both *DmCPD* and *DmGA20ox*, related to brassinosteroids and gibberellins biosynthesis, respectively [74]. Overexpression of *MtDREB1C*, isolated from *Medicago truncatula*, conferred freezing tolerance to China rose without causing any morphological abnormalities [75], and blue chrysanthemum was developed through the introduction of two genes encoding anthocyanin 3′ 5′-*O*-glucosyltransferase (*A3′5′GT*) and flavonoid 3′ 5′ hydroxylase (*F3′5′H*) into the host plant [76]. Thus, it would also be of great value to check phenotypic alterations reported by previous functional genetic studies.

## 5. Conclusions

For ornamental plants, many studies have been attempted to develop new traits. Among them, genome editing is a potential method for improving traits easily and rapidly compared to conventional breeding. The genome-editing system has become a current molecular tool of choice for functional genomics and for developing new traits. Various examples have been reported that the editing system can be connected to an unprecedented acceptance of plant biology and crop yield improvement through fast and targeted mutagenesis and associated breeding [77,78]. Owing to their numerous properties, including easiness, productivity, high specificity, and amenability to multiplexing, genome-editing technologies are revolutionizing crop breeding and paving a new road for breeding techniques. It is obvious that genome editing is an excellent approach to improve the traits of existing floricultural crops and also enables us the chance to expand the usage of ornamental plants for benefits at the industrial or governmental level. In 2016, the United States Department of Agriculture (USDA) succeeded in editing the gene for the common white button mushroom (*Agaricus bisporus*) using the CRISPR-Cas9 system, and the U.S. has decided not to consider it as a GM organism (GMO) [79]. In addition, the Japanese government defined genome-edited end products generated by SDN-1 type modification (mutation without using a DNA template) as not representing GMOs [80]. Until now, however, it is not clear whether other countries will give the same pass to the genome-edited crops/end products, since each country has different GMO policies. Ultimately, with regard to floricultural science, the combination of conventional breeding and genome-editing strategies will be of help in improving the quality of human life through the production of more valuable ornamental flowers.

## Figures and Tables

**Table 1:** Comparison of clustered regularly interspaced short palindromic repeat (CRISPR) genome-editing systems using base editor and prime editor.

	Base Editor	Prime Editor
Fusion components	*nCas9* or *dCas9* + deaminase	*nCas9* + RT
Possible modifications	Transition mutations	All precise modifications
Advantages	Higher editing efficiency, fewer indel byproducts	More targeting flexibility, greater editing precision
Drawbacks	Bystander editing, genome-wide off-targets	Potential transcriptomic dysregulation

*nCas9*: Cas9 nickase; *dCas9*: catalytically inactive Cas9; *RT*: reverse transcriptase.

**Table 2:** List of recent successes in clustered regularly interspaced short palindromic repeats–CRISPR-associated (CRISPR-Cas) systems of ornamental plants.

Species	Material	Targeted Gene	Method	Gene Function	Reference
*Dendrobium officinale*	Protocorm	*C3H*, *C4H*, *4CL*, *CCR*, *IRX*	*Agrobacterium*-mediated transformation	Lignocellulose biosynthesis	[62]
*Ipomoea nil*	Immature embryo	*InDFR*	*Agrobacterium*-mediated transformation	Anthocyanin biosynthesis and white flowers	[55]
*Ipomoea nil*	Immature embryo	*InCCD4*	*Agrobacterium*-mediated transformation	Altered petal color	[56]
*Ipomoea nil*	Immature embryo	*EPH1*	*Agrobacterium*-mediated transformation	Petal senescence	[53]
*Lilium longiflorum,* *Lilium pumilum*	Embryogenic callus	*LpPDS*	*Agrobacterium*-mediated transformation	Albino phenotype	[58]
*Petunia hybrida*	Protoplast	*PhNR*	PEG-mediated protoplast transfection	Deficiency in nitrate assimilation	[48]
*Petunia hybrida*	Leaf	*PhPDS*	*Agrobacterium*-mediated transformation	Albino phenotype	[49]
*Petunia hybrida*	Protoplast	*PhACO1, PhACO2, PhACO3*	PEG-mediated protoplast transfection	Petal senescence	[52]
*Petunia inflata*	Leaf	*PiSSK1*	*Agrobacterium*-mediated transformation	Self-incompatibility	[50]
*Phalaenopsis equestris*	Protocorm	*MADS*	*Agrobacterium*-mediated transformation	Floral initiation and development	[64]
*Torenia fournieri*	Leaf	*F3H*	*Agrobacterium*-mediated transformation	Flavonoid biosynthesis	[57]

*C3H*: coumarate 3-hydroxylase; *C4H*: cinnamate 4-hydroxylase; *4CL*: 4-coumarate:coenzyme A ligase; *CCR*: cinnamoyl coenzyme A reductase; *IRX*: irregular xylem 5; *DFR*: dihydroflavonol-4-reductase; *CCD4*: carotenoid cleavage dioxygenase 4; *EPH1*: EPHEMERAL1; *PDS*: phytoene desaturase; *NR*: nitrate reductase; *ACO*: 1-aminocyclopropane-1-carboxylic acid; *SSK1*: S-locus F-box-interacting SKP1-like 1; *F3H*: flavanone 3-hydroxylase.
